# Case report: Report of a rare encounter: metastasis of renal cell carcinoma to the thyroid

**DOI:** 10.3389/fonc.2024.1350043

**Published:** 2024-04-23

**Authors:** Siyi Xu, Jiawei Xu, Chengdong Yu, Ying Zeng, Lei Tang, Mu Tang, Tenghua Yu, Zhengkui Sun, Xiaofang Zhang

**Affiliations:** ^1^Department of pathology, Jiangxi Cancer Hospital, Nanchang, China; ^2^Jiangxi Medical College, Nanchang University, Nanchang, China

**Keywords:** renal cell carcinoma, thyroid, metastasis, pathology, diagnosis, case report

## Abstract

Renal cell carcinoma (RCC) is the most common renal tumor, with lung, bone, and liver being the primary sites of metastasis. Thyroid metastasis, on the other hand, is relatively uncommon. Metastatic tumors in the thyroid gland typically manifest as multiple or isolated nodules, which can be easily overlooked due to the lack of specific clinical and imaging features. However, the identification of thyroid metastasis suggests the presence of systemic metastasis and is indicative of a poor prognosis for patients. In this paper, we present two cases of thyroid metastasis following nephrectomy, with the objective of enhancing understanding among medical community regarding the diagnosis and treatment of thyroid metastasis originating from renal cell carcinoma. By raising awareness about this phenomenon, we emphasize the importance of early detection and diagnosis to improve patient prognoses. The implementation of standardized treatment protocols at the earliest possible stage is also emphasized. Through this research, we aim to contribute to the early identification and management of thyroid metastasis in patients with renal cell carcinoma, ultimately leading to improved outcomes.

## Case 1

**Figure 1:**
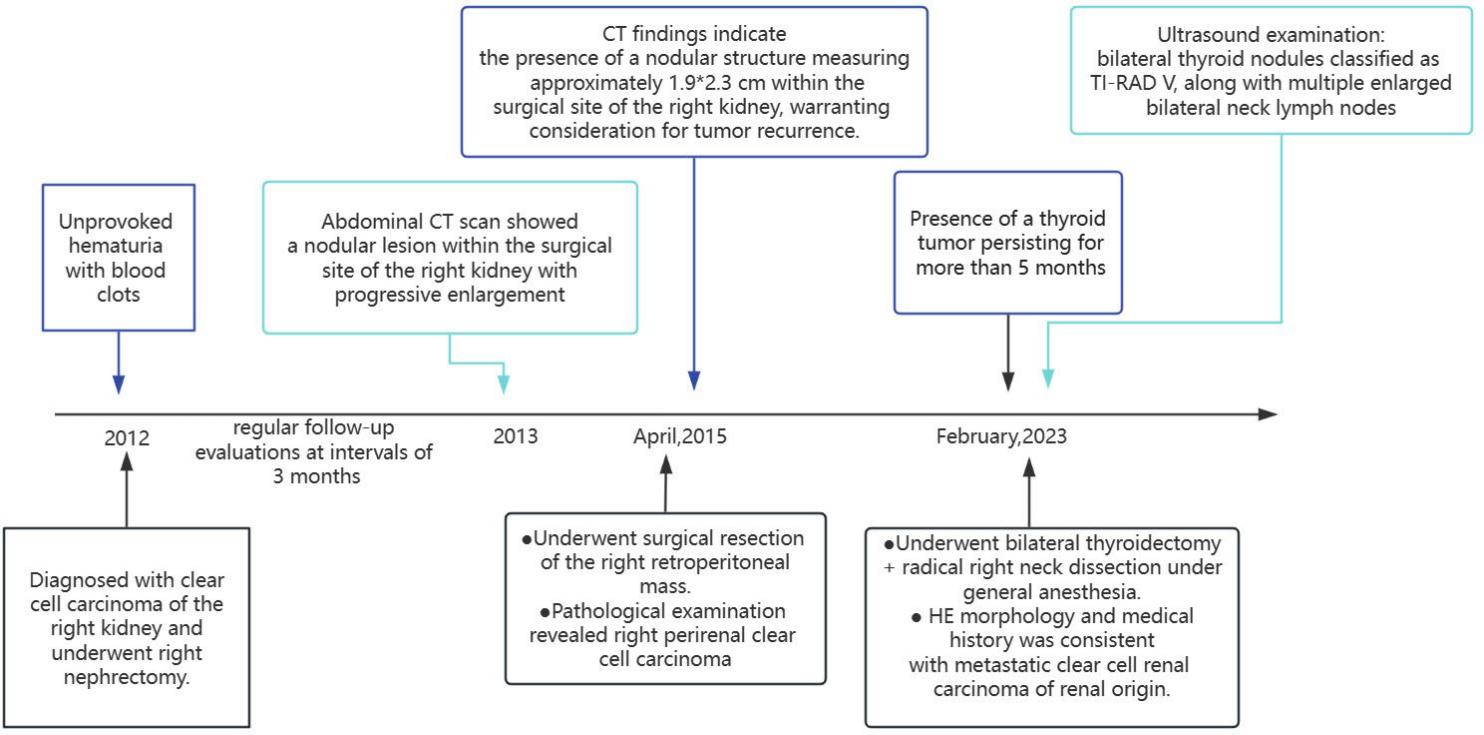
Patient medical history timeline of case 1.

In 2012, a middle-aged gentleman of 48 years presented with the clinical manifestation of hematuria characterized by the presence of blood clots, without an identifiable underlying etiology (**Figure 1**). Seeking medical attention, he promptly sought care at a local healthcare facility, where a comprehensive abdominal computed tomography (CT) examination revealed findings indicative of a neoplastic growth localized within the right renal structure. Following comprehensive preoperative evaluations, a right nephrectomy was conducted at the hospital. The subsequent postoperative histopathological examination revealed clear cell carcinoma originating from the right kidney. The postoperative patient underwent regular follow-up evaluations at intervals of 3 months. Approximately 1 year post-surgery, a subsequent abdominal CT scan revealed the presence of a nodular lesion within the surgical site of the right kidney, the nature of which remained undetermined. Consequently, ongoing surveillance and close monitoring were advised. Following the initial evaluation, subsequent reviews conducted in March revealed a progressive enlargement of the nodule within the surgical site of the right kidney. Further assessment at the Affiliated Cancer Hospital of Fudan University in April 2015 demonstrated a significant enlargement of the nodule to 24 mm, thereby raising concerns regarding the potential presence of recurrent foci. Our CT findings indicate the presence of a nodular structure measuring approximately 1.9*2.3 cm within the surgical site of the right kidney, warranting consideration for tumor recurrence. On April 24, 2015, the patient underwent surgical resection of the right retroperitoneal mass. The postoperative pathological examination revealed right perirenal clear cell carcinoma, Furman grade 3, characterized by small nuclei, abundant and translucent cytoplasm, lamellar arrangement, and infiltrative growth of tumor cells (immunohistochemistry was not performed at that time). The patient had a favorable postoperative recovery.

**Figure 2:**
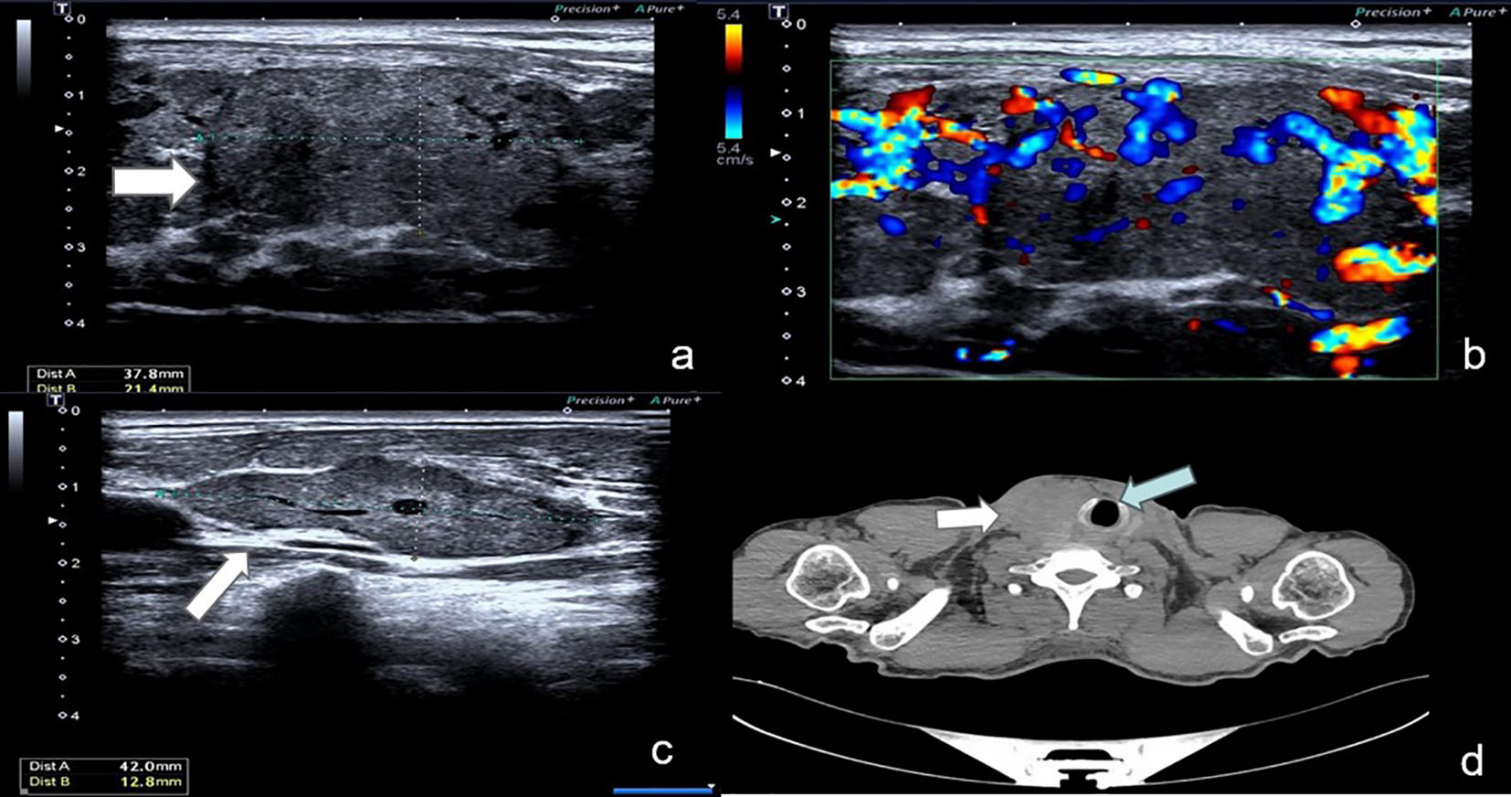
Ultrasound and CT of the thyroid gland and neck. **(A, B)** Multiple hypoechoic nodules were identified in the right lobe of the thyroid gland, some of which were partially fused, forming a mass with a maximum dimension of approximately 38*21mm. The nodules exhibited irregular morphology and were associated with abundant blood flow signals. **(C)** Multiple enlarged lymph nodes were detected in the right cervical region, the largest of which was about 42*13mm and contained abundant blood flow signals. **(D)** CT shows that the right lobe of the thyroid gland exhibited enlargement, accompanied by the presence of a slightly hypodense mass measuring approximately 4.3*4.9 cm. The surrounding fat interstitial space appeared blurred, and the lesion showed indistinct demarcation from the adjacent trachea and esophagus, resulting in leftward displacement of the trachea.

**Figure 3:**
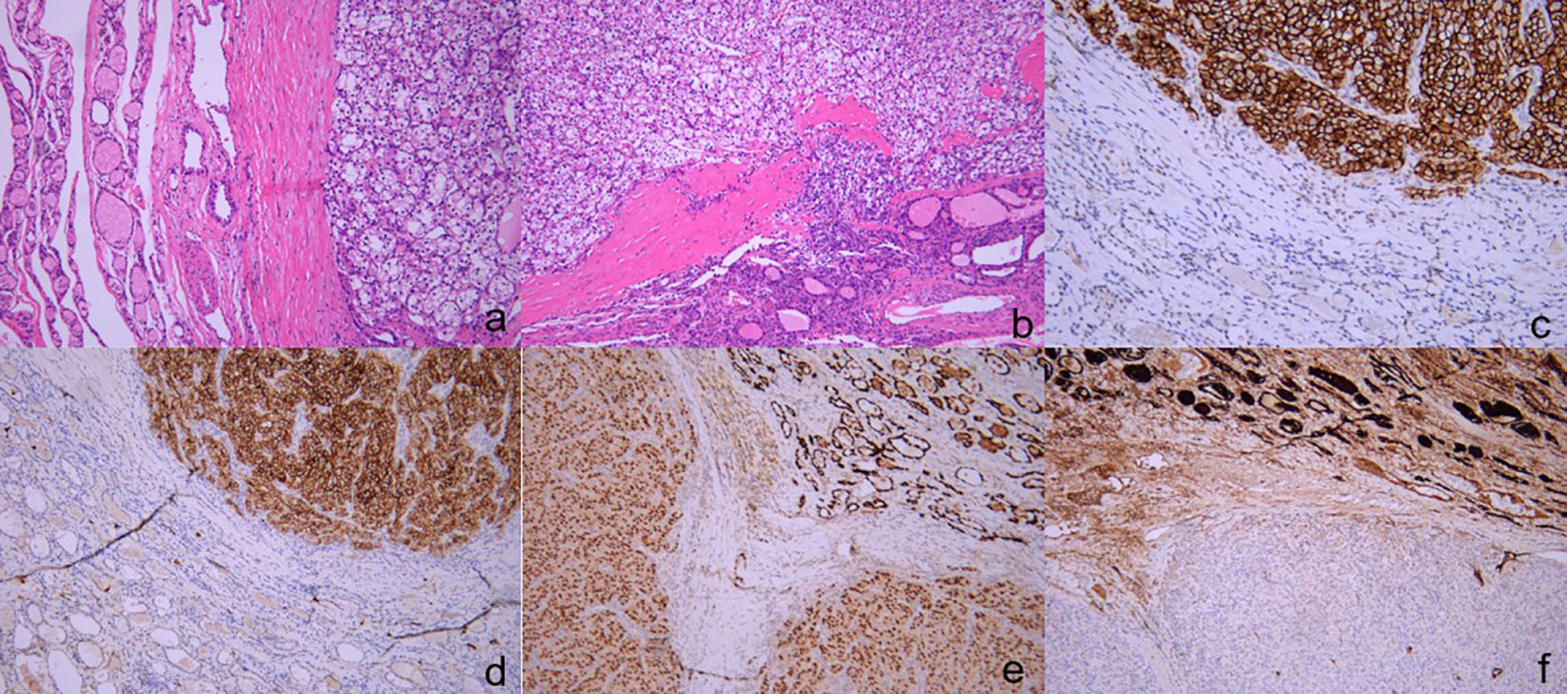
The pathology and immunohistochemical staining of thyroid specimens. The tumor tissue demonstrates a distinctive organization in nested clusters and sheets, characterized by the presence of large nuclei with intense staining. (**(A)** H&E, ×200), (**(B)** H&E, ×100). Immunohistochemical staining revealed that the tissue were positive for CA9 (**(C)** ×200)[CD10 (**(D)** ×200)[PAX-8 (**(E)** ×100), negative for TG (**(F)** ×100).

In February 2023, the patient underwent an ultrasound examination due to the presence of a thyroid tumor persisting for more than 5 months. The findings revealed bilateral thyroid nodules classified as TI-RAD V, along with multiple enlarged bilateral neck lymph nodes. Physical examination: The neck exhibited symmetrical features and had a soft texture, there was no jugular vein distention, and the trachea was centrally aligned, palpation of the neck revealed a palpable swelling in the right thyroid gland, measuring approximately 3.5 cm. The swelling had a soft consistency and did not elicit any discomfort upon pressure. It exhibited mobility and moved up and down with swallowing. No evident abnormalities were detected in the left thyroid gland. Additionally, there were no palpable enlarged lymph nodes in the bilateral neck, and no tremor or vascular murmur was observed. Ultrasound examination of the thyroid gland and neck (**Figure 2**): Multiple thyroid nodules were identified, classified as C-TIRADS class 4A, within the right lobe of the thyroid gland, multiple hypoechoic nodules were diffusely distributed, some of which partially merged to form a mass, the largest nodule measured approximately 38*21 mm, displaying an irregular morphology with a well-defined boundary, abundant blood flow signals were detected within. In the left lobe and isthmus, multiple hypoechoic nodules were also identified, the largest nodule in this region measured approximately 21*8 mm, featuring an irregular morphology with a clear boundary and abundant blood flow signals, furthermore, multiple enlarged lymph nodes were detected in the right side of the neck. Chest and abdominal CT scan (**Figure 2D**): the scan revealed an occupying lesion in the right lobe of the thyroid gland, warranting further ultrasonography, suspiciously enlarged lymph nodes were observed in the right upper mediastinal trachea, multiple nodules were also present in both lungs, indicating the possibility of metastases, postoperative changes were noted in the left kidney. The blood thyrotropin level showed a mild elevation at 4.817 uIU/mL, while the blood levels of FT3, FT4, and cancer markers were all within the normal range. Given the patient’s prior history of renal carcinoma and local recurrence, an ultrasound-guided fine-needle aspiration (FNA) of the thyroid mass was performed as part of the preoperative evaluation, to our surprise, the cytologic examination yielded results indicative of follicular carcinoma of the thyroid. The patient underwent bilateral thyroidectomy+ radical right neck dissection under general anesthesia on February 15, 2023. During the intraoperative examination, multiple nodules were identified in the right thyroid gland, with the largest measuring approximately 3*3 cm. The nodules had a firm consistency, and the capsule remained intact. Subsequent postoperative pathological analysis revealed the presence of several gray-white masses in the right lobe of the thyroid gland, which were fused together and measured approximately 5*4*2.5 cm, additionally, gray-yellow medium-sized nodules, approximately 1.6*1.5*0.6 cm in size, were observed in the left lobe and isthmus near the envelope, the tumor tissue exhibited cluster and sheet arrangements of cancer nests, characterized by large, deeply stained nuclei. Furthermore, lymph node metastases were detected in 6 out of 11 lymph nodes examined. Immunohistochemical results (**Figure 3**): CD10(+), Pax-8(+), CK19(+), CyclinD1(+), Ki-67(+, 15%), TG(-), TPO(-), TTF-1(-), Cal-3(-), CD56(-), CgA(-), Syn(-), TFE3(-), BRAF-V600E(-) the combination of HE morphology and medical history was consistent with metastatic clear cell renal cell carcinoma of renal origin. The patient underwent regular post-operative evaluations at the hospital, revealing no evidence of disease progression up to the present moment.

## Case 2

**Figure 4:**
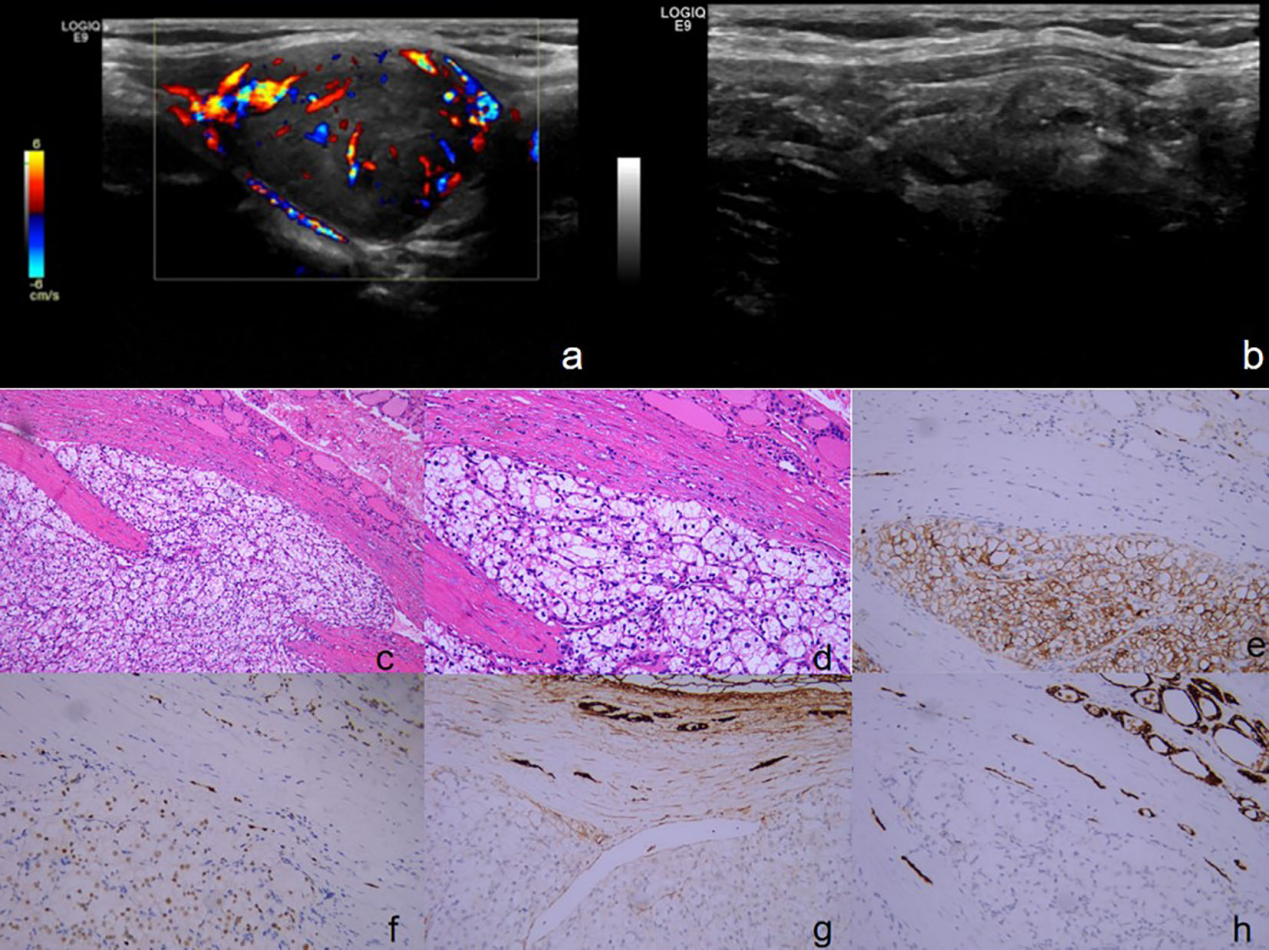
**(A, B)** Ultrasound of the thyroid and neck: **(A)** The largest mixed echogenic nodule in the right lobe of the thyroid gland, measuring about 27*20 mm, with clear borders, regular morphology, and showed abundant blood flow signals both within and around the area. **(B)** Multiple mixed echogenic nodules were observed in the right lobe of the thyroid gland, and some of the nodules exhibited punctate strong echoes. **(C-H)** The pathology and immunohistochemical staining of tyroid specimens. Tumor cells exhibit a growth pattern characterized by the formation of nests composed of cells with translucent cytoplasm, the nuclei of these cells are rounded, displaying varying sizes and prominent staining, with abundant interstitial blood sinuses. (**(C)** H&E, ×100), (**(D)** H&E, ×200). Immunohistochemical staining revealed that the tissue were positive for CD10 (**(E)** ×200)[PAX-8 (**(F)** ×200)[, negative for TG (**(G)** ×200)[TPO (**(H)** ×200).

A 62-year-old female was admitted to our institution in 2021 due to the incidental discovery of an anterior neck mass that had been present for 1 year. She reported a history of right nephrectomy for clear cell carcinoma of the right kidney at an external hospital, with satisfactory postoperative recovery and regular follow-up. Approximately one year ago, she noticed a painless lump in the anterior neck measuring approximately 2.0*1.5 cm. She did not experience any associated symptoms such as pain, fever, sweating, dysphagia, or hoarseness, and she did not seek medical attention at that time. There was no family history of kidney tumors or thyroid disease. Physical examination: Multiple palpable swellings were identified in the bilateral thyroid gland and isthmus, the largest swelling measured approximately 2.5 * 2.0 cm and exhibited a smooth surface with medium consistency, no pressure pain was reported, and the boundaries of the swellings remained clear, the swellings moved up and down with swallowing, and no vascular murmurs were detected. Ultrasound of the thyroid and neck (**Figure 4**): The ultrasound examination revealed the presence of multiple mixed echogenic nodules (TI-RADS III) in the right lobe of the thyroid gland, these nodules exhibited well-defined borders, regular morphology, and prominent blood flow signals within and surrounding them, some of the nodules exhibited punctiform strong echogenic clusters. Thyroid function indices were found to be within normal limits. The patient underwent a right-sided thyroid lobectomy combined with a left-sided thyroid lumpectomy on June 17, 2021. Postoperative pathological findings revealed the presence of a clear cell tumor in the right lobe of the thyroid gland. The cut surface of the right thyroid gland exhibited a grayish-yellow area with dimensions measuring approximately 2.2 * 1.5 * 2 cm. The mass demonstrated a well-defined boundary and was encapsulated. Furthermore, a cancerous thrombus was observed within the choroid. In contrast, the mass identified in the left lobe of the thyroid gland was determined to be a nodular goiter. Immunohistochemistry (**Figure 4C–H**): TG(mild+), TPO(-), CK19(-), CyclinD1(+), TTF-1(-), Ki-67(+,10%), pan-CK(+), CD10(+), CD31(-), CD34(-), EMA(-), PAX-8(+), Cal-3(-), CgA(-), P504s(+). The combination of morphological characteristics and immunohistochemical analysis provided evidence consistent with the diagnosis of metastatic clear cell renal carcinoma.

Furthermore, abdominal MRI revealed the presence of multiple blood-rich occupying lesions in the pancreas caput and pancreas cauda, indicating a high probability of neuroendocrine tumors. Given the patient’s medical history and the findings from this examination, a consultation with a medical oncologist recommended that the patient be referred to the medical oncology department for further treatment. However, despite these recommendations, the patient and her family declined any therapeutic interventions. Following a satisfactory recovery, the patient was discharged from the hospital. Through a subsequent telephone follow-up, it was ascertained that the patient is currently experiencing a favorable recovery trajectory.

## Discussion

This article presents a case report of two patients with thyroid metastasis originating from clear cell renal carcinoma. The clinical features, imaging findings, and treatment approaches for thyroid metastasis from renal clear cell carcinoma are discussed and summarized in the context of relevant literature (**Table 1**). The aim is to provide valuable insights for clinicians in the diagnosis and management of this rare condition.

**Table 1:** Cases of tumor-to-tumor metastasis from RCC to thyroid carcinoma.

Study	Age	Time interval to diagnosis of metastatic disease(year)	FNAB	Localization	Treatment	IHC
C.Gawlik et al (1)	63	1	Accepted, indeterminacy	Isthmus + Unilateral	Left thyroid lobectomy and isthmectomy	PAX-8(+),TTF-1(+)
A.Solmaz et al (2)	64	1.5	Not accepted	Unilateral	total thyroidectomy	CD10(+),Vimentin(+),TTF-1(-)
D.Macedo-Alves et al. (3)	80	9	Accepted, indeterminate	Unilateral	Hemithyroidectomy and isthmectomy	CD10(+),Vimentin(+),TTF-1(-), TG(-),chromogranin(-)
I. Chiardi et al. (4)	76	8	Accepted, suspicious	entire thyroid gland	thyroidectomy	
J. L. Shi et al. (5)	56	5			resection of thyroid metastatic renal cell carcinoma	Rcc (+), AmACR (−),vimentin (+++), CK (−), TPO (−), CK19 (−), galectim-3 (+++) and CD10 (++)
C.P.Ramírez-Plaza et al. (6)	62	Thyroid nodules as the first symptom	Accepted, indeterminate	Bilateral	total thyroidectomy with a prophylactic central compartment dissection	CD10(+),TTF-1(-),TG(-),
C.E.Connolly et al. (7)	84	Thyroid nodules as the first symptom	Not accepted	entire thyroid gland	total thyroidectomy	
F.Badawi et al. (8)	63	14	Accepted, indeterminate	Bilateral	total thyroidectomy	CD10(+), CAIX(+),galectin-3(+), vimentin(+)
M.Yamauchi et al. (9)	59	Thyroid nodules as the first symptom	Accepted, indeterminate	Unilateral	left hemithyroidectomy	CD10(+)
M. Di Furia et al. (10)	53	1	Accepted, indeterminate	Unilateral	total thyroidectomy	RCC(+),CD10(+), Vimentin(+), TTF1(-), Thyroglobulin(-)
R.N.Hellums et al. (11)	69	10	Accepted, diagnosed	Unilateral	hemithyroidectomy	CA9(+),PAX8(+), TTF-1(+)
F. Wu et al. (12)	55	4	Accepted, indeterminate	Bilateral	total thyroidectomy+ post-operative chemotherapy	RCC(+),CD10(+),PAX-8(+), TTF1(-), Thyroglobulin(-)
M. Kefeli et al. (13)	80	18	Accepted, unreported	Unilateral	left hemithyroidectomy	PAX-8(+),CD10(+),Vimentin(+), TTF1(-),TG(-),GATA-3(-)
C. Manini et al. (14)	42	synchronic	Accepted, indeterminate	Unilateral	total thyroidectomy	PAX-8(+),CD10(+),carbonic anhydrase IX(+)
F. Medas et al. (15)	62	6	Not accepted	Unilateral	total thyroidectomy	CD10(+),TTF1(-),Hector Battifora mesothelial cell monoclonal antibody-1(-),galectin-3(-)

+: weakly positive, ++: moderately positive, +++: strongly positive, -: negative.

Renal cell carcinoma (RCC) is a prevalent malignancy in the field of urology, representing approximately 2%-3% of adult malignant tumors. Among the various histological subtypes, clear cell renal carcinoma (ccRCC) is the predominant type, constituting approximately 70%-80% of primary renal tumors (8, 16). The onset of RCC is characterized by its subtle and indolent nature. Astonishingly, approximately 20%-30% of patients present with distant metastases upon the initial diagnosis, while an additional 30% develop heterotopic metastases even after undergoing radical surgery. Regrettably, the prognosis for metastatic kidney cancer remains disheartening, with a meager median survival of merely 2 years (17). The lungs, bones, liver, brain, and contralateral kidneys are the most frequent sites of metastasis for RCC, while other less frequently observed sites of metastasis have also been reported in isolated cases.

Despite the thyroid being an organ with abundant vascularization, the occurrence of metastatic tumors from distant sites to the thyroid is exceedingly uncommon, constituting a mere 1.4%-3% of all malignant thyroid tumors (16). Several scholars postulate that the rarity of metastatic tumors in the thyroid can be attributed to two factors. Firstly, the normal thyroid tissue is highly vascularized but lacks the capacity for cancer cell entrapment. Consequently, cancer cells find it challenging to establish a foothold in this environment. Secondly, the elevated levels of iodine and oxygen within the thyroid gland create an inhospitable milieu for tumor cell proliferation, thereby exerting a certain degree of growth inhibition (18). The median interval for the development of thyroid metastasis in renal clear cell carcinoma has been reported to be 8.7 years. However, the presence of thyroid metastases signifies an advanced stage of the primary tumor. Furthermore, a significant proportion of patients (35-80%) with thyroid involvement also exhibit metastases in multiple organs, which is associated with an unfavorable prognosis (19).

Thyroid metastases from RCC typically manifest as solitary or multiple thyroid masses. The presence and severity of symptoms such as dysphagia, dyspnea, and hoarseness depend on the size and location of the metastatic masses. In the cases presented in this study, both patients exhibited neck masses with localized lesions. However, these masses did not lead to concurrent symptoms such as dyspnea and were non-specific when compared to primary thyroid tumors.

In general, imaging studies alone are insufficient to differentiate between primary and secondary tumors of the thyroid. Debnam et al (20) reported that secondary thyroid tumors, both primary and metastatic, exhibit similar ultrasound characteristics to primary thyroid tumors. They can be classified into nodular and diffuse infiltrative changes, with nodular patterns accounting for approximately 65% of cases. Nodular secondary thyroid tumors often manifest as solid hypoechoic nodules with ill-defined margins, accompanied by hyperechoic foci and detectable blood flow signals. On the other hand, the diffuse infiltrative pattern is nonspecific and cannot be reliably distinguished from thyroiditis or other diffuse thyroid diseases. In patients with a history of ccRCC, long-term follow-up and routine thyroid ultrasound should be performed, and metastasis should be highly suspected if new thyroid nodules are detected (21). Compared to CT, PET-CT exhibits higher sensitivity in tumor diagnosis, making it an advantageous imaging modality for detecting tumor recurrence and metastasis. PET-CT provides whole-body imaging, which is particularly useful for patients with a previous history of malignant tumors. When evaluating the thyroid gland with 18F-PET-CT, the presence of a mass or diffuse uptake or infiltration should raise a high suspicion of thyroid metastasis (22). Furthermore, in the diagnosis of secondary thyroid tumors, a non-functional or “cold” nodule observed on thyroid scintigraphy can provide valuable diagnostic information (23). Given that PET-CT and scintigraphy are not standard procedures at our institution and are associated with considerable costs, both patients opted against undergoing these tests due to financial constraints.

Although fine-needle aspiration (FNA) is commonly employed in the preoperative assessment of thyroid nodules, its diagnostic accuracy for metastatic thyroid tumors, particularly from renal cell carcinoma (RCC), has certain limitations. Studies have reported a false-negative rate of up to 28.7% in the diagnosis of RCC metastases using FNA. This highlights the challenges associated with accurately identifying metastatic thyroid tumors originating from RCC through FNA alone (24). Given the patient’s previous history of renal tumor and recurrence in Case 1, a preoperative ultrasound-guided fine needle aspiration (FNA) was performed on the thyroid mass. However, the cytologic results indicated follicular carcinoma of the thyroid, highlighting the limitations and uncertainty of FNA in accurately diagnosing secondary thyroid tumors. Histopathological examination using Hematoxylin and Eosin (HE) staining of postoperative resection specimens provides a basis for distinguishing between primary and secondary thyroid tumors. However, it is worth noting that identifying tumors secondary to clear cell renal carcinoma and breast carcinoma can be challenging due to their tendency to exhibit follicular glandular structures that closely resemble those composed of clear cells, which is a common occurrence in hyperplastic thyroid nodules (25). For cases where differentiation between primary and secondary thyroid tumors is challenging using HE staining alone, additional immunohistochemical staining (IHC) can be employed. IHC not only aids in distinguishing between primary and secondary thyroid tumors but also assists in identifying the origin of secondary tumors. Positive staining for markers such as thyroglobulin (Tg), calcitonin, and thyroid transcription factor (TTF-1) suggests primary thyroid tumors, while negative staining for these thyroid markers suggests secondary thyroid tumors. In the case of secondary thyroid tumors originating from renal cell carcinoma (RCC), positive staining for markers such as CD10[PAX-8/PAX-2[CA9[CK7[vimentin can aid in the diagnosis, while GATA-3, ER, and PR, aiding in the diagnosis of breast cancer-derived tumors (26).

The management of secondary thyroid tumors encompasses various treatment modalities, including surgery, chemotherapy, and radiation therapy. Typically, distant metastases from malignant tumors do not warrant surgical intervention. However, in cases where patients present with isolated metastatic thyroid nodules, surgical treatment may offer the potential for long-term control or even cure. The 5-year overall survival rate in such cases typically ranges from 30% to 50%. Surgical resection of isolated metastatic thyroid nodules has shown promise in achieving favorable outcomes for selected patients (27). It has been posited that total thyroidectomy is recommended for patients lacking concurrent metastases from other anatomical sites, in order to optimize the management of symptoms stemming from neck metastases of RCC and achieve complete excision of metastatic lesions (28). Russell et al. (29) found that significantly improved overall survival rates among patients with secondary thyroid tumors who underwent surgical intervention as opposed to conservative treatment. The greatest surgical benefit was observed in cases of kidney cancer, with a median survival of 6 months for patients receiving conservative treatment compared to 27 months for those who underwent surgery. However, the survival benefit associated with surgery was found to be lower for tumors originating from the lung or gastrointestinal tract, potentially due to the aggressive nature of these primary tumors. It is important to note that prompt surgical intervention is recommended when thyroid tumor patients experience compression symptoms, as it can effectively enhance their quality of life. For patients with extensive systemic metastases or thyroid metastases that cannot be completely resected, chemotherapy or radiation therapy may be considered as alternative treatment options. Compared with other metastatic tumors, RCC demonstrates limited sensitivity to chemotherapy. Therefore, the cornerstone of current RCC treatment lies in targeted therapy and immunotherapy. Among these, the targeted drug sunitinib has proven to be highly effective in the management of metastatic RCC, and the findings from the CheckMate 9ER study (30) provide compelling evidence that a combination approach involving targeted therapy with cabozantinib and immunotherapy utilizing nivolumab outperforms sunitinib in both efficacy and safety. Additionally, various alternative combination regimens are currently under investigation in clinical trials, offering promising prospects for improved prognoses among patients with metastatic RCC. RCC inherently exhibits resistance to radiotherapy, making postoperative radiotherapy generally an unadvised course of action (31). Neither of the two patients described in this article underwent postoperative adjuvant therapy, continuing treatment with sunitinib targeted therapy or immunotherapy would likely be more beneficial for them.

Both patients featured in this report developed thyroid metastases subsequent to undergoing nephrectomy surgery (Case 1 exhibited metastases nine years after the RCC diagnosis, while the precise timing of metastasis onset in Case 2 remains unknown). Both patients underwent comprehensive preoperative assessments as part of the standard protocol preceding surgery. The treatment approach adopted aligns with the established management principles for thyroid metastases originating from RCC, emphasizing surgical intervention. However, it is noteworthy that postoperative adjuvant therapy was not pursued, representing a potential area for improvement in the treatment protocol. Based on the preoperative evaluation of the cases, neither clinical presentation nor imaging modalities could effectively discriminate between primary thyroid cancer and metastatic thyroid tumors. Despite encountering a considerable number of false negatives, preoperative FNA remains indispensable in the diagnostic process, the diagnosis of metastatic thyroid tumors originating from RCC continues to depend on postoperative morphology and immunohistochemical identification. Given the current progress in Radiomics and Pathomics, we envisage the potential for training artificial intelligence (AI) models to distinguish between primary and metastatic thyroid cancer, as well as metastatic thyroid tumors, preoperatively. However, this endeavor demands a significant repository of image data sourced from imaging and pathology, underscoring the importance of enhanced collaboration among clinical researchers to facilitate data sharing for effective AI model development. In line with the cases presented in this study, the primary treatment modality for patients with thyroid metastases originating from RCC remains surgical intervention. While surgery alone can confer a degree of survival benefit, contemporary therapeutic approaches such as targeted therapy and immunotherapy have demonstrated efficacy. Enhanced outcomes may be achievable by maintaining and intensifying targeted or immunotherapeutic interventions following surgery. Further research endeavors can be directed towards identifying targets for RCC metastasis, thereby enhancing the efficacy of metastasis control strategies in RCC management.

Thyroid metastases originating from renal cell carcinoma (RCC) are infrequent in clinical practice, and the current literature mainly consists of sporadic case reports with a dearth of standardized guidelines for clinical management. Differentiating secondary thyroid tumors from primary thyroid tumors can be challenging as they lack specific clinical manifestations and imaging features. Therefore, a comprehensive evaluation of the patient’s clinical presentation, medical history, and imaging findings is crucial for accurate diagnosis. Immunohistochemical staining plays a pivotal role in confirming the diagnosis. Surgical intervention is the primary treatment modality and has shown significant improvements in overall patient survival. However, further research and consensus guidelines are needed to establish a standardized approach for managing thyroid metastases from RCC. This article offers clinical practitioners valuable insights by detailing the diagnostic and treatment experiences of two patients with thyroid metastasis originating from renal cancer. Moreover, it conducts a comprehensive literature review to consolidate and critically assess the deficiencies encountered throughout the diagnostic and treatment procedures. The objective is to provide a substantive reference for healthcare professionals.

## Data availability statement

The original contributions presented in the study are included in the article/supplementary material. Further inquiries can be directed to the corresponding authors.

## Ethics statement

The studies involving humans were approved by Ethics Committee of Jiangxi Cancer Hospital. The studies were conducted in accordance with the local legislation and institutional requirements. The participants provided their written informed consent to participate in this study. Written informed consent was obtained from the individual(s) for the publication of any potentially identifiable images or data included in this article.

## Author contributions

SX: Writing – review & editing, Writing – original draft. JX: Writing – review & editing, Writing – original draft. CY: Writing – review & editing, Writing – original draft. YZ: Writing – review & editing, Writing – original draft. LT: Writing – review & editing, Methodology. MT: Writing – review & editing, Methodology. TY: Writing – review & editing, Supervision. ZS: Writing – review & editing, Supervision. XZ: Writing – review & editing, Supervision, Methodology.
